# Investigating face processing in online interactions via UK–US hyperscanning using fNIRS

**DOI:** 10.1162/IMAG.a.1101

**Published:** 2026-01-23

**Authors:** Uzair Hakim, Jack Adam Noah, Xian Zhang, Natalie Gunasekara, Antonia Hamilton, Paola Pinti, Ilias Tachtsidis, Joy Hirsch

**Affiliations:** Department of Medical Physics and Biomedical Engineering, UCL, London, United Kingdom; Department of Psychiatry, Yale School of Medicine, New Haven, CT, United States; Institute of Cognitive Neuroscience, UCL, London, United Kingdom; Birkbeck University, London, United Kingdom; Department of Neuroscience, Yale School of Medicine, New Haven, CT, United States; Department of Comparative Medicine, Yale School of Medicine, New Haven, CT, United States; Wu Tsai Institute, Yale University, New Haven, CT, United States

**Keywords:** hyperscanning, face processing, online communication, fNIRS, coherence, videoconferencing

## Abstract

Videoconferencing technology has become a staple of everyday life and has found widespread use in business, education, and tele-medicine. Despite this, there have been few empirical studies investigating the neural correlates of interactions during this form of communication. This study investigates the neural mechanisms of face processing during online videoconferencing, employing functional near-infrared spectroscopy (fNIRS). We synchronised presentation and acquisition of fNIRS data across laboratories in the UK and US using custom Python software and a third-party computer system. Using this framework, we examine how different presentations of faces (live-online vs. static images) influence social cognition and inter-brain coupling (IBC) in a videoconferencing context. Forty participants (20 dyads) engaged in online sessions where they viewed either dynamic video feeds or static images of their partners’ faces. Our findings did not show preferential activity in the right supramarginal gyrus during the observation of live on-line faces as expected from prior studies of live in-person face gaze. However, occipital–temporal and dorsal visual streams were more active for live-online faces than static-online faces. There was no observed evidence for increased cross-brain synchrony during the live face condition compared with the static face condition. Findings suggest that on-line studies of social interaction open a novel field of investigation that is differentiated from live interactions.

## 1 Introduction

Videoconferencing technology has become a staple of everyday life, its use is common in education, business, healthcare and personal use. There is a recent body of literature evaluating this new form of communication and the perceived differences occurring between videoconferencing and “in-person” communication, perhaps the most marked being “Zoom fatigue” (Bailenson, 2021; Fauville et al., 2021; Nesher Shoshan & Wehrt, 2022; Ratan et al., 2022). One primary difference of communicating via videoconferencing, compared with “in-person” is that videoconferencing allows a live presentation of the partners face (live-online) or this can be turned off and replaced with a static image of the same person (static-image). In this paper we will examine the differences between these two forms of face presentation within the context of non-verbal online videoconferencing. Throughout the paper we will use the term videoconferencing to refer to conditions which take place through Zoom or similar online communication tools and in-person to refer to conditions which take place whilst participants are physically co-present. When referring to a face that is presented in real time to a participant (i.e. not a recording), we will use the term live. We will use the term live-online to refer to conditions where a real-time face is presented to a participant through Zoom and static-online where a still image is presented.

Faces have been shown to be processed differently from objects in behavioural studies (Behrmann et al., 1992; Bruce et al., 1991; Damasio et al., 1990; Tanaka & Farah, 1993; Yin, 1969). This has also been shown with neuroimaging studies using static representations of faces, where specific regions including the fusiform gyrus, amygdala, superior temporal gyrus, and orbitofrontal cortices have been consistently found to be active during static face observation (Haxby et al., 2000; Kanwisher et al., 1997; Zhen et al., 2013). These findings have been further developed with the inclusion of dynamic face movements (Allison et al., 2000; Fox et al., 2008; Haxby et al., 2000; Puce et al., 1998, 1999), finding increased activation in the supramarginal, superior temporal gyri, referred to as the lateral stream of face processing, during the observation of videos of faces relative to static faces. Electrophysiological findings from monkeys during face processing confirm the homologous specificity for static face processing in the ventral temporal lobe. These findings suggest that single cells in the ventral stream are also face selective (Chang & Tsao, 2017; Freiwald & Tsao, 2014; McMahon et al., 2014), and include similarities with human fMRI findings as well as preference for a direct face gaze (Iidaka et al., 2012). Together, these findings suggest that two distinct visual streams, the ventral stream for processing static, invariant features of faces and the lateral stream for processing dynamic information obtained from observing moving faces (Bernstein et al., 2018; Bernstein & Yovel, 2015; Freiwald et al., 2016; Haxby et al., 2002; O’Toole et al., 2002) encode face information. Importantly, the studies mentioned here have focused on static pictures and pre-recorded videos, or static pictures (e.g. Puce et al., 1998). Here we focus on live-online vs. static-online face.

Advances in the application of functional near-infrared spectroscopy (fNIRS) have allowed neuroimaging data to be acquired from the full head of participants, whilst providing a more ecologically valid experimental environment. In turn, this provides a route to further advance the understanding of face processing by allowing participants to interact with a physically present partner. This form of face presentation differs to the previously mentioned studies because they can offer reciprocal interactions during an exchange and can be considered as primary sources of social information. As such recent work using fNIRS has focused on the “dyad” to probe neural underpinnings of live face processing (Cañigueral et al., 2021; Descorbeth et al., 2020; Dravida et al., 2020; Hirsch et al., 2017, 2021, 2022; Kelley et al., 2021; Noah et al., 2017, 2020; Zhao et al., 2023). These studies find increased neural activation occurring in the right temporal–parietal junction (rTPJ) during viewing of live faces relative to static faces, recorded videos and human-like robot faces. The rTPJ has been associated with mentalising and social communication (Carter & Huettel, 2013; Frith & Frith, 2021), and its activation suggests that live faces indeed engage higher order social cognition systems. In particular, several studies (Hirsch et al., 2017, 2022; Kelley et al., 2021; Noah et al., 2020; Zhao et al., 2023) have assessed social interaction using passive face-gaze as the form of social interaction between participants. The significance of live faces as socially salient stimuli is advanced by the Interactive Brain Hypothesis (De Jaegher et al., 2016; Di Paolo & De Jaegher, 2012) which proposes that live social interaction drives dynamic neural activity that either does not occur or is less prominent during similar non-interactive tasks (De Jaegher et al., 2010). Taking this theoretical framework and the findings from the studies mentioned above together, passive face gaze at a live face can be considered as an elementary form of interaction.

The benefit of fNIRS to allow participants to interact with each other in real time has extended the paradigm of neuroscience experiments by allowing the simultaneous acquisition of neuroimaging data whilst participants are engaged with each other, known as *hyperscanning* (Czeszumski et al., 2020; Hakim et al., 2023; Montague et al., 2002). The acquisition of neuroimaging data from multiple participants allows experimenters to examine the Inter-Brain Coupling (IBC) between participants whilst they interact. IBC is the extent to which the neural responses between two regions of interacting brains are synchronised. IBC is often referred to as “coherence” or “neural coupling.” Although a consistent theoretical framework for the interpretation of IBC has yet to be established, it has been proposed to reflect sharing of information (Hasson & Frith, 2016), mutual prediction (Hamilton, 2021; Kingsbury & Hong, 2020), or perhaps more ambitiously, some level of social connectedness (Hoehl et al., 2021).

Despite the uncertainty of the theoretical framework, prior studies examining IBC have found increased IBC in the TPJ when participants interact with each other compared with acting alone (Nguyen et al., 2021; Szymanski et al., 2017), suggesting a link between behaviour of participants and IBC. Thus it is important to understand the extent to which partners are interacting or not when interpreting IBC. For example, interpersonal synchronisation also includes behavioural effects such as spontaneously generated body movements that occur only under conditions where participants are able to view each other (Koul et al., 2023). Behavioural contingency during videoconferencing has been examined in various contexts. Myers et al. (2024) found that the ability of a partner to respond to an infant in video chat interactions predicted joint visual attention, whereas constraints such as across-screen references and increased interaction complexity reduced engagement. These findings highlight the importance of considering how the limitations of videoconferencing (including restricted gaze coordination) impact IBC. Further, Roche et al. (2022) focused on affect during videoconferencing, demonstrating that real-time responsiveness in gaze and affect predicts positive social engagement, further highlighting the importance of the impact videoconferencing has on eye contact and, therefore, social contingency. Language learning in toddlers was investigated over both videoconferencing and in-person conditions, finding that responsive video chat did not fully support toddlers during word learning (Troseth et al., 2018), emphasising that responsiveness alone is not always sufficient in digital contexts. These studies collectively underscore the impact of videoconferencing on behavioural contingency, which in turn suggests an impact on IBC.

In this study, we focus on face processing during videoconferencing, examining the differences between live-online and static-online conditions. The examination of IBC with respect to face processing has previously compared live-in-person faces to static pictures and pre-recorded videos (Hirsch et al., 2017; Noah et al., 2020), between high and low eye-contact conditions (Dravida et al., 2020) as well as between live-in-person and pre-recorded videos for Typically Developed, TD, and Individuals with Autism Spectrum Disorder, (ASD) participants (Hirsch et al., 2022). In the case of Hirsch et al. (2017) and Noah et al. (2020) the live-in-person conditions showed higher IBC than the alternatives. Similarly, Dravida et al. (2020) found increased IBC during the high eye contact conditions and Hirsch et al. (2022) also found the live-in-person conditions for TD participants gave the highest IBC values. In each instance, the Wavelet Transform Coherence, WTC, was computed between regions commonly associated with social cognition. Noah et al. (2020) computed it between the Angular Gyrus, Hirsch et al. (2017) between the MTG and STG, and Dravida et al. (2020) between the Occipitotemporal cortex and the MTG. Taken together, these findings suggest that viewing live faces, as opposed to pre-recorded videos, or static images increases the degree of IBC that is recorded between interacting participants. An important consideration with face processing in-person, compared with videoconferencing is the differences in eye contact. As reviewed in Bohannon et al. (2013), when communicating using videoconferencing, people overwhelmingly prefer to use camera-screen configurations which allow eye contact, and find the typical downward gaze prevalent in videoconferencing to be ambiguous due to reduced eye contact. Bohannon et al. (2013) have shown that misalignment of gaze in videoconferencing disrupts natural eye contact, leading to reduced communication efficiency, trust, and impressions of partners. This suggests that limitations in gaze perception can impact both IBC and neural activity during face processing online, possibly because of reduced joint attention. In this study, we use a standard camera-screen configuration such that the gaze of participants is downcast to examine gaze in an ecologically valid videoconferencing situation.

Face processing thus far has been investigated using dynamic faces (video recordings presented on a screen, which are not live) and live-in-person (using real people). Videoconferencing technologies offer a third route for face processing. Faces are presented on a screen but are live and responsive, hereafter referred to as live-online. The relationship between this form of face processing and current thinking of face processing systems is not established.

The examination of IBC in relation to videoconferencing methods has been hindered due to methodological limitations. Many laboratories investigating IBC do so using a single fNIRS system, with optodes divided between participants, as a result both participants must sit in the same room. This offers an economically advantageous and easy-to-use approach to run the experiment, at a cost of reduced spatial resolution. Specifically in relation to examining IBC in videoconference settings, an additional limitation is the requirement to keep participants in proximity. To ensure participants are in fact remote from each other, they would have to be housed in separate structures, or kept in different rooms (Balters et al., 2020).

To date, there have been three studies using fNIRS which have examined social cognition when using videoconferencing technology (Balters, Miller, Li, et al., 2023; Balters, Miller, & Reiss, 2023; Zhao et al., 2023). Each study compared videoconferencing conditions with in-person conditions and examined IBC using WTC. These three studies differ in two ways. Firstly, the protocol employed by Balters et al. (2023), Balters, Miller and Reiss (2023), and Zhao et al. (2023) differs in how the participants interact with each other. Balters et al. (2023) and Balters, Miller, and Reiss (2023) ask participants to take part in tasks where they actively work with their partner to solve problems, create and express emotions to each other, through verbal and non-verbal communication. In contrast, Zhao et al. (2023) simplified the task and asked participants to passively gaze at the face of the partner, displaying a live video feed of their partners face during the videoconferencing condition, and the live in-person face picture.

Analysis of single-brain data assessing differences between videoconferencing and in-person conditions differs between these studies. Balters et al. (2023) and Balters, Miller, and Reiss (2023) did not find differences in activation between the two forms of communication. In contrast, Zhao et al. (2023) found increased activity in the right supramarginal gyrus (rSMG) during the “real in-person” face viewing. The difference between the findings of Zhao et al. (2023) and Balters et al. (2023) could be due to the degree of interaction between the participants. The participants in Balters et al. (2023) interacted with each other in a multi-modal way, with participants able to verbally communicate, gesture, and express themselves in relation to the task at hand. Thus, the increased amount of socially salient information available to participants in each condition could contribute to the similarity in neural processing between the different forms of communication. In contrast the passive face gaze task employed by Zhao et al. (2023) is a more elementary form of interaction, where social information is sparser. As mentioned by the authors, the reduced perception of dynamic social cues such as facial micromovements likely disrupted the neural processes that are present during in-person interactions.

Furthermore, Zhao et al. (2023) found increased IBC between the somatosensory association cortex (SSAC) of participants during the in-person condition compared with the virtual. In agreement, Balters et al. (2023) also found increased IBC in prefrontal and temporal regions associated with social cognition during in-person socio-emotional tasks and found altered IBC pathways during problem solving and creativity tasks; however, in the follow-up paper from Balters, Miller, and Reiss (2023), the authors found that expressing appreciation could reduce the differences in IBC between conditions. One possible explanation for the reduction in IBC across studies is a general weaker alignment of social cues between participants during videoconferencing, including facial micromovements and a reduced ability to establish co-ordinated behaviours.

Zhao et al. (2023) hypothesised that the “real in-person” face would activate areas associated with social cognition, including the rTPJ, more so than the same, live face presented through a screen (live-online). To assess this, the authors employed a multi-modal experimental setup, with dual EEG & fNIRS and eye-tracking information from both participants engaged in the task. Their findings support the hypothesis. Behaviourally, the authors found increased duration of eye contact, and pupil diameter during the in-person face presentation compared with the videoconferencing condition. This was consolidated further with findings from the EEG data showing reduced theta-band oscillations during the videoconferencing condition, suggesting an early frequency band separation of faces presented using this medium compared with in-person. fNIRS findings were consistent with both visual-sensing and EEG findings, showing reduced activity in temporoparietal regions. The authors suggest that in-person interactions allow participants to better detect subtle micromovements in the face and eyes. With the implementation of videoconferencing tools however, these are subdued, leading to a reduction in IBC as well as less activity in the SMG, in essence, during the in-person condition participants are better able to detect dynamic social cues than during the videoconferencing condition.

In this study we build on the prior findings from Zhao et al. (2023). Here we isolate the dynamic component of the live-online face to determine whether the presence of dynamic social cues alone elicits neural activity in social regions such as the supramarginal gyrus, SMG, and IBC between the angular gyrus, AG, of the participants. Following the same language as Zhao et al. (2023), we use the term interaction to refer to mutual passive face gaze. Based on the findings from Zhao et al. (2023), we hypothesise (1) that there will be no difference in brain activity in the rSMG between the live-online and static-online conditions because the on-line stimulus conditions compromise the fine-tuned micromovements of the live in-person face and (2) that the Inter Brain Connectivity, IBC, between the AG of participants during the live-online condition will be increased relative to the static-online condition. This hypothesis is due to the increased sharing of information (as proposed by Hasson & Frith (2016)) in the live online face condition relative to the static-online face condition. A prior study (Noah et al., 2020) has reported an increase in IBC between the angular gyri of the participants engaged in mutual face-gaze interactions suggesting that this region may be specialised for computations that integrate rapid and fine-tuned micromovements of facial dynamics.

In this study, we present a method to synchronously acquire neuroimaging data from remote laboratories to investigate live social interactions in the online world. We conduct an investigation of the neural basis of face processing during online non-verbal interactions via videoconferencing. To do this, we connect two laboratories over Zoom whilst synchronously recording neural activity during passive face gaze conditions using fNIRS. We investigate passive face processing during online non-verbal interactions, varying between live-online (viewing the live video of their partners face) and static-online (viewing a picture of their partners face). Hypotheses comparing these conditions are based on findings from Zhao et al. (2023), namely, their finding suggesting that live-online faces, when presented on a screen, do not elicit the same activity as would be expected from a real, physically present face. As an exploratory approach, we hypothesise that the live-online condition will activate social regions not activated by the static-online condition and that there will be no significant difference in activity in the right SMG when participants view the live-online face or the static-online face. Furthermore, considering these recent inconsistent findings of IBC during online communications, in-person dynamic faces, and static faces, we compare the IBC between the Angular Gyri of interacting participants under the two online conditions of this investigation, on-line live face and on-line static face.

## 2 Methods

### 2.1 Participants

In total, 40 self-declared healthy participants (20 dyads) participated in the study, 26 female, 11 male and 3 self-classified “others”; mean age: 28.2 +/- 8; age range from 18 to 50 years. All 40 participants were included in the analysis. Recruitment was conducted separately in the UK and the US according to institutional guidance. Participants were paired together in order of recruitment, and not matched for any demographics. Prior to enrolment, participants were asked whether they had any neuropsychiatric conditions.

The Yale experimental site was the Brain Function Laboratory at the Yale School of Medicine (300 George Street, Suite 902, New Haven, CT, 06511). The 20 participants recruited in the US had previously demonstrated reliable activation in the primary motor cortex during a finger tapping task. The UCL experimental site was the Institute of Cognitive Neuroscience (Alexandra House, 17-19 Queen Square, London WC1N 3AZ). The 20 participants recruited in the UK were recruited through a database and advertisements on social media, and posters placed around campus. All cross-Atlantic participants were strangers to each other and were assigned on-line partners in order of recruitment. All participants provided written informed consent in accordance with institutional guidance. Ethics approval was granted by the Yale University Human Investigation Committee (HIC#: 1501015178) and the UCL Research Ethics Committee (5975/003).

### 2.2 Experimental paradigm

This task is an online adaptation of the study from Hirsch et al. (2017). In the original paradigm, participants observed either real in-person live face, or a static image of a person’s face. Here we adapted this, so participants observed either their partners live-online face over Zoom, or a static image of their partners face. Counterbalancing was conducted by computing all possible orders of condition presentation, these were then randomly assigned to each dyad. Participants were given identical instructions at the start of the experiment. Both participants were instructed to gaze passively at the face of their partner and to avoid speaking and large head movements to preserve data quality, no specific instructions were given in relation to eye contact or facial expressions.

**Fig. 1.**
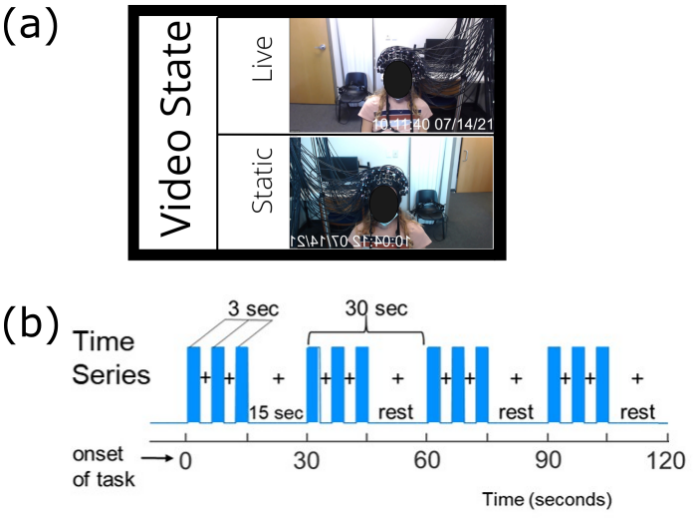
(a) Experimental conditions. Each cell is one experimental condition that was tested. Video state refers to whether the video feed from the webcam or a static photograph taken before the experiment began is shown. (b) The time series for each experimental condition. Stimulus (the partners face) is presented for 3 s periods lasting 18 s, followed by 12 s of rest (fixation cross). Each condition lasts 2 min in total.

These alterations in facial representation provide two conditions to be tested as illustrated in Figure 1a. Task blocks were 15 s long, with partner faces being presented for multiple 3 s blocks as illustrated (Fig. 1b) and were co-occurring for all participants within a dyad. The 3 s time period was chosen for participant comfort due to the difficulty of maintaining eye contact for longer than 3 s. This was alternated with a 15 s baseline/rest block (Fig. 1b). Each experimental condition lasted a total of 2 min (Fig. 1b), and repeated twice. The experimental paradigm was coded in PsychoPy (Peirce et al., 2019). To remove the Zoom window during the static experimental runs, a grey PsychoPy window was generated and maximised in front of the Zoom window.

Faces were presented on 24-inch screens in both laboratories, and participants were positioned approximately 70 cm from the screen to ensure the visual angles of the observed faces were the same at both sites. The Logitech C920 HD webcam was used at both laboratories. To ensure the static faces were comparable with the live-online faces, still images were acquired just before data collection, after the fNIRS equipment had been set up. Two pictures were acquired, one with the face mask on and one without. The images were acquired at the same location and position as the Zoom call to ensure comparable sizes of faces were presented. During the Zoom call, participants self-view was turned off and the background was the view of the room. Calls were maximised so the entire screen was filled with the call.

### 2.3 Data acquisition

**Fig. 2.**
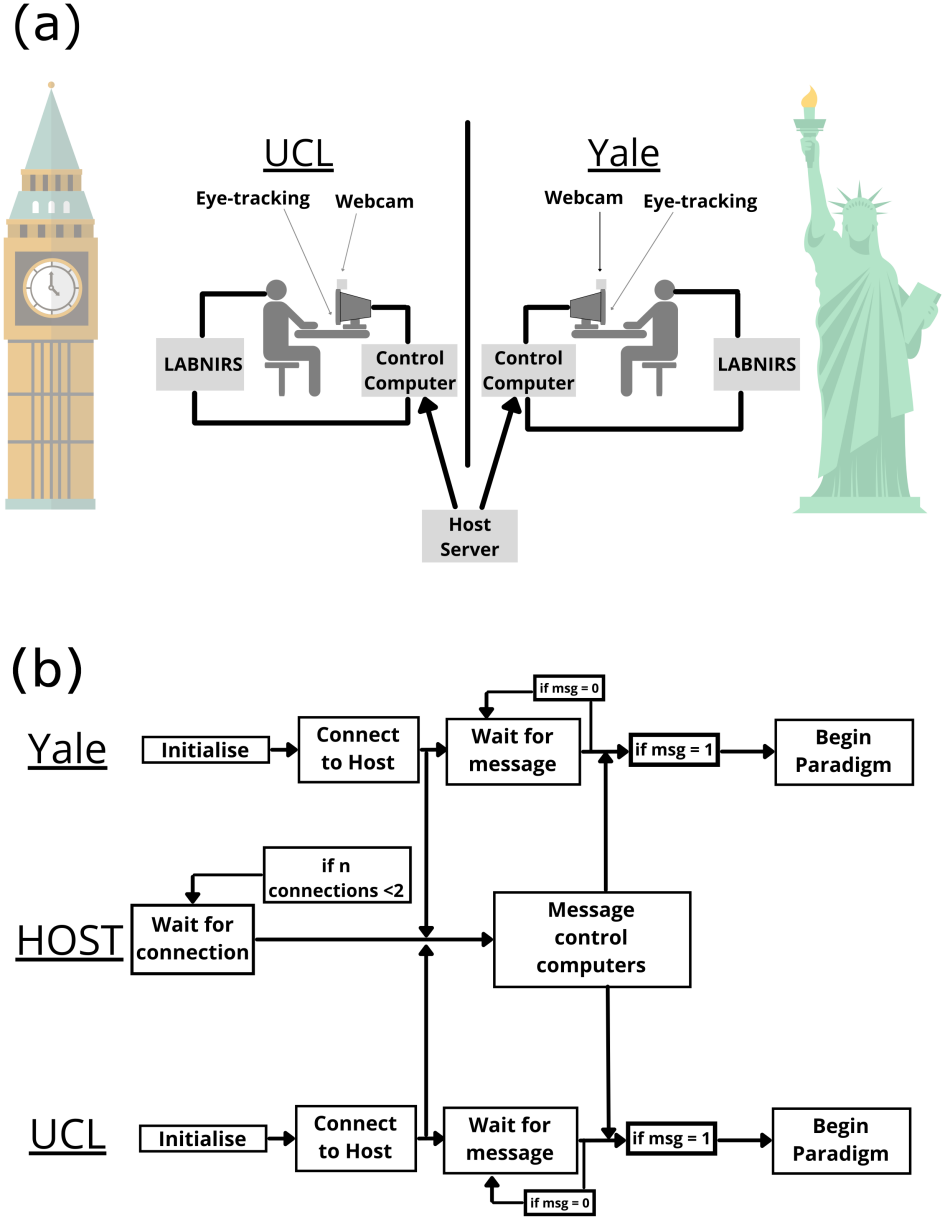
(a) Experimental setups at both sites. Each site uses the Shimadzu LABNIRS system. UCL has 88 channels, whilst Yale has 134 channels. Eye tracking at UCL is recorded using the Tobii Eyetracker 5 with custom code to obtain data, whilst Yale uses the Tobii Pro x3-120 with data obtained using the Tobii official software. (b) The pipeline for how laboratories are synchronised. Client-side code is run at both sites which initialises the paradigm software (PsychoPy, Psychtoolbox etc). Once initialised, client attempts to connect to the Host server. Once host has received connections from both clients, a trigger is sent to both clients to start the paradigm. The paradigm code maintains control over starting and stopping of the LABNIRS.

Data were acquired synchronously at UCL and Yale sites. The same measures were acquired at both sites. The experimental setup at both sites is shown in Figure 2a, whilst the synchronous connection method is shown in Figure 2b.

#### 2.3.1 Functional near-infrared spectroscopy

Haemodynamic brain data were acquired at both laboratories using Shimadzu LABNIRS systems. The LABNIRS systems emit NIR light at three wavelengths, 780, 805, and 830 nm. The system used at Yale consisted of 40 source–detector pairs, constituting 134 channels covering the entire head, whereas the system at UCL used 28 source–detector pairs, constituting 88 channels covering the bilateral temporal, parietal, and occipital regions. Source and detectors were placed 3 cm apart at UCL, whilst Yale used distances of 3 cm for participants with a head circumference greater than 56.5 cm, or 2.75 cm for a head circumference less than 56.5 cm. These differences were accounted for during the computation of the concentrations using the modified Beer–Lambert Law. Data were sampled at 8.13 Hz at Yale and 12.34 Hz at UCL. Caps were positioned using anatomical landmarks based on the 10–20 system. At UCL, the top of the cap was positioned at Cz of the participant, whilst at Yale the anterior midline optode holder was positioned ~2cm above the nasion. Hair was removed from the optode holders using a lighted optic-probe prior to inserting optodes, to ensure NIR light was not obstructed by hair. Sufficient optode–scalp coupling was determined by the presence of heart beats in the concentration traces.

#### 2.3.2 Eye tracking

Eye movements were acquired at both sites using Tobii eye trackers. Yale used the Tobii Pro x3-120 which samples eye movements at 120 Hz. At UCL the Tobii 5 eye-tracker, sampling eye-movements at 133 Hz, was used. The data from the eye trackers were not used for the analyses presented in this paper.

#### 2.3.3 Optode localisation

Registration of optodes to brain anatomy at UCL was achieved by an electromagnetic digitiser (*Polhemus Liberty, n.d.*) used to record optode coordinates and the location of five anatomical locations (nasion, inion, right and left auricular points, and vertex) for each participant in 3D space. A photogrammetry method was used at Yale where the same five anatomical locations were marked on the fNIRS cap using green stickers. A Structure IO 3D camera was then used to construct a 3D model of the participants head with the fNIRS cap on. Optode locations were then manually marked in MATLAB and 3D coordinates obtained using Fieldtrip (Oostenveld et al., 2010). In both cases, Montreal Neurological Institute (MNI) coordinates were obtained using the NIRS-SPM toolbox (Ye et al., 2009). Anatomical correlates were estimated with the TD-ICBM152 atlas using WFU PickAtlas (Maldjian et al., 2003).

Due to channel configuration differences between the laboratories, a spatial smoothing procedure using a Gaussian-weighted kernel was used to co-localise channel configurations between laboratories. Pairwise distances between measurement channels and a pre-defined spatial mask were computed. To maintain maximal spatial resolution, the pre-defined spatial mask was the ideal optode placement for the Yale configuration. A Gaussian kernel was then applied where the weight of each element in the kernel reduces exponentially with distance, controlled by a smoothing parameter which determines the extent of influence. The formula for the kernel computation is provided in Eq. 1, where σ controls the smoothing parameter and d_i,j_ refers to the distance between channel i of the participant and channel j of the mask. For this analysis, the value was set to 0.01 to ensure localised smoothing whilst preserving spatial specificity, allowing nearby channels to contribute to the signal whilst minimising the influence of distant channels.

$$K_{ij}=\exp\left(-\frac{d_{ij}^{2}}{\sigma }\right).$$ (1)

To ensure adequate normalisation, each column of the kernel is divided by its sum, preserving the relative signal amplitudes. This method redistributes local signals whilst maintaining spatial specificity. After this procedure was applied, data from both laboratories were output as 134 channels, allowing analysis to be conducted concurrently with both laboratories. At locations, channels were not present for UCL, the data were extremely small in amplitude, and would not contribute to the statistical analysis.

### 2.4 Inter-laboratory synchronisation

The method developed to connect the laboratories at UCL and Yale together consists of three hardware components: (1) a server, (2) client-UCL, and (3) client-Yale. At each component, a script is run that connects client-UCL and client-Yale together. All scripts were coded in Python, and makes use of the Python socket module, part of the Python standard library. This serves the dual function of ensuring that the code is open source and implementable, and also that the experiment being conducted can be managed within the same code. The method uses the server as a handshake to align the time that the paradigms are presented, and equipment starts recording. A flowchart showing how the method works is given in Figure 2b.

Briefly, each laboratory runs their paradigm code, containing the socket-connection code which connects independently to the server. Once the server has received connection messages from both laboratories, it relays a message to all connected clients which acts as a starting signal for the experiment to begin. Our method maintains logs of all events at both sites. Computer clock times are acquired using the Python time module and are recorded when (1) code was run, (2) connection to server was made, (3) message from the server received, (4) start time of the experiment, (5) start of each task/rest block, and (6) end of experiment, these are output to a text file which is stored at each client and on the server. This allows us to ensure that each experiment is running synchronously. We first determined the ground clock-difference between laboratory computers, and found a 0.5 s delay, using this we were able to ensure that any significant deviations from this difference in our logs were investigated and any data exempted from the analysis.

In order to validate this connection method, 200 connect–disconnect tests were run. The times of each connection were recorded through the client-side code and analysed to evaluate timing differences between computer clocks. Underlying differences in the computer clocks varied on a day-to-day basis because of other uses of the PCs by each laboratory, and so to ensure that differences in recordings were because of lags in the code and not clock times, a video call was started where the clock times of each participant was overlayed on the screen. Screen recordings were acquired using ffmpeg (*FFmpeg*, n.d.) which captured the Zoom call, showing the partners’ computer clock, and the researchers’ computer with the time in the taskbar. These were then used to determine the “ground truth” time difference. Once established, the difference between the connection time for the Yale computer clock and the UCL computer clock was subtracted from each other, taking into consideration the ground truth time difference. This methodology was applied to evaluate the consistency of the initial connection, as well as the presentation of stimulus blocks. Results from this analysis are provided in Supplementary Materials in Figures S1 and S2.

### 2.5 Data analysis

All data analyses were conducted using MATLAB R2019a.

#### 2.5.1 Data exclusions

fNIRS data were visually inspected to ensure adequate data quality. The frequency spectra of raw intensity data were inspected to observe the heart beat component occurring at approximately 1 Hz (Yücel et al., 2021). Following this, concentration data were inspected to ensure a physiologically correct haemodynamic response. Concentration data from channels with poor scalp coupling were identified by having perfectly mirrored HbO_2_ and HHb, over-saturations, and no heart beat component. The 5% of channels were removed (across all participants) using this criterion. Following this, an automated method identified signals where the root mean square of the raw data was more than 10 times the RMS of the average signal. The two methods displayed concordant results and no participants were fully excluded.

#### 2.5.2 fNIRS pre-processing

All fNIRS pre-processing was carried out using the same pre-processing pipeline, with the same code base. Because data were acquired at different sampling rates, the data acquired from UCL at 12.34 Hz were resampled to match the Yale sampling rate using the *resample* function in MATLAB. All further pre-processing and subsequent analyses were conducted using the upsampled data.

fNIRS data were converted from optical densities to HbO_2_ and HHb using the modified Beer–Lambert Law (Matcher et al., 1995) using the Shimadzu LABNIRS in-built software. HbO_2_ and HHb data were then band-pass filtered using a 4^th^ Order Butterworth filter with cut-offs at 0.01–0.2 Hz (Pinti et al., 2019). The filter parameters were constructed and then applied using the *butter* and *filtfilt* functions in MATLAB, respectively. The Global Mean Removal algorithm, a principal component analysis spatial filter (Dravida et al., 2017; Noah et al., 2021; Zhang et al., 2016), was used to remove systemic components of the data assumed to be non-neuronal in origin (Tachtsidis & Scholkmann, 2016).

#### 2.5.4 Functional activation analysis

The General Linear Model (GLM) was used to evaluate functional activation in each individual participant brain. In all analyses, the signal employed was the difference between the oxy and deoxy signals known as Hb_Diff_ (Hakim et al., 2022; Kaynezhad et al., 2019; Kolyva et al., 2014; Tachtsidis et al., 2009). The canonical haemodynamic response function (HRF) was used as the regressor for the analysis used to evaluate contrast effects based on the comparison of our experimental conditions. The canonical HRF was formed using SPM8 (Friston, 2003) and was convolved with a boxcar signal (consisting of 15 s active +15 s rest blocks) to generate the model HRF used in the analysis. Beta values (regression coefficients) representing the goodness of fit of the model to the measured fNIRS data were computed using the ordinary least squares (OLS) method. Group results based on these beta values were rendered on a standard MNI brain template using the NIRS-SPM software (Ye et al., 2009). Beta values were used in t-tests to determine differences between conditions using SPM8.

#### 2.5.5 Inter-brain coupling analysis

IBC was computed based on the Wavelet Coherence method (Cui et al., 2012; Grinsted et al., 2004) as described previously (Dravida et al., 2020; Hirsch et al., 2017, 2018, 2022; Noah et al., 2020). This is a symmetric time–frequency analysis which computes the common power of input signals at specific frequencies. This form of analysis is particularly suited to our experimental paradigm since intrinsic leader–follower roles were not present, and the symmetric nature of the wavelet coherence method is able to elucidate any increases in IBC that may be present. For this analysis, we focused on the HHb signal since HHb is less contaminated by systemic noise (Hakim et al., 2022; Kirilina et al., 2012), and the possibility of spurious IBC as a result of noise is an issue in the computation of the IBC (Burgess, 2013). The basis wavelet used was a complex Gaussian provided by the MATLAB Wavelet toolbox. The number of octaves was four, and the range of frequencies was between 0.4 and 0.03 Hz, corresponding to periods between 2.5 and approximately 30 s. The number of voices per octave was also four. In total, 16 “scales” were used, in increments of 2.5 s. Channels were grouped into regions of interest (ROI) based on shared anatomy, this allowed the IBC between differing optode configurations to be computed whilst optimising the signal-to-noise ratio (SNR). Grouping was based on the identification of 14 bilateral regions. (1) Angular Gyrus (BA39); (2) dorsolateral prefrontal cortex (BA9); (3) dorsolateral prefrontal cortex (BA 46); (4) pars triangularis (BA 45); (5) supramarginal gyrus (BA40); (6) middle temporal gyrus (BA21); (7) superior temporal gyrus (BA22); (8) somatosensory cortex (BA1, 2, and 3); (9)somatosensory association cortex (BA7); (10) pre-motor and supplementary motor cortex (BA6); (11) subcentral area (BA43); (12) inferior frontal gyrus (BA47); (13) visual cortex (Area V3, BA19), and (14) frontal eye fields (BA8). This was conducted for participants within a dyad as well as for shuffled dyads, in keeping with the standard for the field. For this study, shuffled dyads consisted of one participant from each laboratory to ensure that the shuffled partners were comparable with the real partners.

The computation of the IBC using the wavelet coherence can be done using either the fNIRS signal itself, which contains the task effect or by using the residuals of the signal, which do not contain the task effect and contain frequency components not related to the physiological components of the fNIRS signal. In this work, we employed the residual method to ensure that common task effects were not included. Using the residuals has its grounding in Psychophysiological Interaction (PPI (O’Reilly et al., 2012)) analysis which has been used to evaluate functional connectivity between remotely located brain regions. To compute the IBC using this method, the GLM analysis was first conducted, where the beta values represent the task component of the signal, the “left-over” residuals essentially reflect the neuronal response not affiliated to any task. Using this method, spontaneous neural responses that are not specifically task driven can be observed and IBC relating to these transient, spontaneous responses was computed using the wavelet transform coherence.

## 3 Results

### 3.1 Functional dyadic activation

Here we present the group results from the GLM analysis, representing the functional activation in individual brains as they observe either live or static variations of their partners. The results shown are based on the HbDiff signal. All conditions were performed on-line using the Zoom platform.

#### 3.1.1 Observing the live face > Observing the static face

**Fig. 3.**
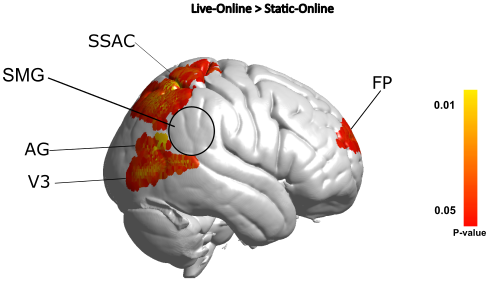
Neural activation clusters of the Live-Online relative to the Static-Online results. Activation less than p < 0.05 seen in the Frontopolar, Somatosensory Association Cortex, Angular Gyrus, and V3 regions. Activation in the SSAC survives FDR correction at p < 0.05. Circle indicates the expected activation based on prior in-person findings.

To test whether the live dynamic presentation of online faces elicits activity in the areas of the brain associated with social interactions as seen previously for in-person studies, we contrasted activity in participants when they observed their partners live face compared with their static face (see Fig. 1a). This analysis compares the live and static conditions. The results are shown in Figure 3 and Table 1. MNI coordinates in Table 1 with a * survive FDR correction at p < 0.05. Neural activity in the Angular Gyrus, V3, frontopolar area, and somatosensory association cortex (BAs 5 and 7) showed increased activity when participants viewed their partners live-online face. Activation occurring in the SSAC (BA7) survives FDR correction at p < 0.05. For comparison, the circle indicates the lateral stream region known to be activated during live in-person conditions (Hirsch et al., 2022; Kelley et al., 2021; Noah et al., 2020). We note it is not significantly activated during the live online condition relative to the static on-line condition.

**Table 1.** Results for the contrast Live-Online > Static-Online.

		Peak voxels							
Contrast	Threshold	MNI	T	P	df	Anatomical regions	Brodmann area	Probability	N voxels
Live-Online > Static-Online	0.05	56 -70 18	3.03	0.00218	39	Angular gyrus	39	62	497
						V3	19	27	
		14 62 30	2.28	0.01423	39	Frontopolar area	10	67	121
		36 -58 66*	3.59	0.00046	39	Somatosensory association cortex	7	69	1492
						Somatosensory association cortex	5	20	

Results are shown for regions showing activation at p < 0.05.

* Regions survive FDR correction at p < 0.05.

### 3.2 Inter-brain coupling, IBC

Here we present the IBC between the angular gyri of the two partners, computed using the wavelet coherence for the live face relative to the static face. This ROI was selected a priori based on prior reports of coherence between this region during in-person face-to-face gaze (Noah et al., 2020).

**Fig. 4.**
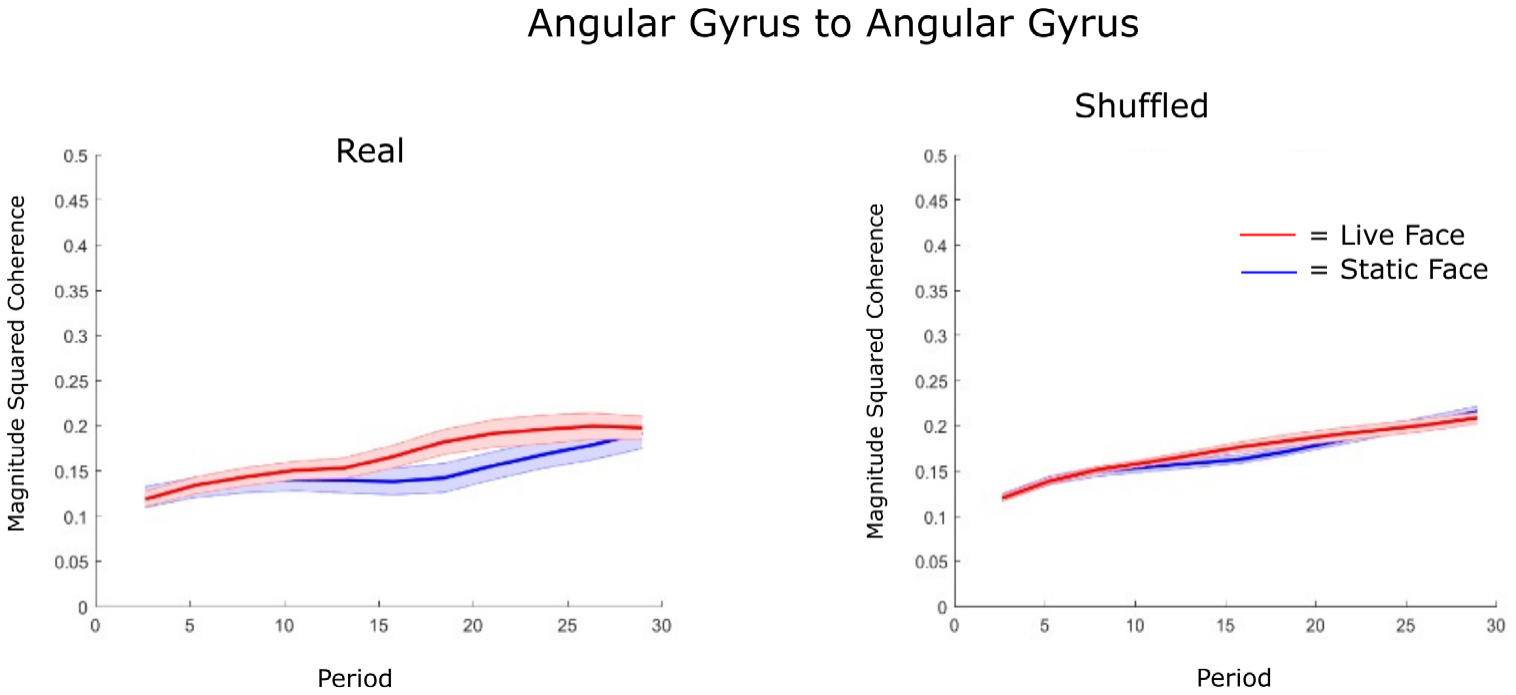
Wavelet coherence reflecting Inter-Brain Coherence between the angular gyri of interacting participants (left) and shuffled participants (computationally paired participants who were not real partners during the experiment) right. The IBC, y-axis, is shown across a range of periods from 5 s to 30 s representing the physiological haemodynamic response function, x-axis. Supplementary Table S1 provides the statistical comparisons for the data over this range of periods. Although visual inspection of the real partner functions (left panel) might suggest an increase in cross-brain coherence for the online live condition, statistical comparison with the control conditions (shuffled partners), ANOVA interaction effects (F(1, 76) = 3.04 (p = 0.09) at the peak of the haemodynamic response function (18. 43 s), fails to support that hypothesis. See Supplementary Table S1.

Figure 4 shows the IBC results for the live face relative to the static face. The magnitude squared coherence (MSC) was computed using the wavelet method and the HHb signal. MSC is displayed on the y-axis, and period (in seconds) on the x-axis. Red and blue lines indicate the mean MSC value for each period for the live and static faces, respectively, with the shading corresponding to the standard deviation. All participant pairs were averaged together for each period. Figure 4 (left) shows the IBC variations for all real dyads, whilst Figure 4 (right) shows the IBC variations for “shuffled pairs.” Shuffled pairs are computed analytically by computing the MSC for pairs other than the real partner.

Although the real on-line partners displayed differences between static-online faces at the 18.4 s period (p < 0.01), this effect was not different from the effect observed for the shuffled partners (p < 0.05). To test whether the real pairs were significantly different from the shuffled pairs, we conducted a two-way ANOVA with two factors: dyad type (real vs. shuffled) and face condition (live vs. static) (Gelman & and Stern, 2006; Nieuwenhuis et al., 2011). At the 18.4 s period, the p-value for the interaction analysis is 0.09. The lowest p-value for the ANOVA occurs at the 42 s period, at 0.05. The full table of results is provided in the Supplementary Materials, Supplementary Table S1.

## 4 Discussion

Motivated by recent findings of altered face processing during videoconferencing conditions (Balters et al., 2020; Balters, Miller, Li, et al., 2023; Zhao et al., 2023), we have conducted an investigation into the neural underpinnings of face processing during online interactions. We have presented results comparing two common forms of facial presentation during online interactions: live-online and static-online faces. Based on previous live in-person studies, we expected to see no difference in activation in the rSMG when participants view the live face compared with the static face. Our findings confirmed this expectation. However, an increase in activity for live (dynamic) on-line faces was observed in dorsal (rSSAC) and ventral (rAG and rV3) regions relative to the static faces, however, only the rSSAC survived FDR correction. We also evaluated levels of IBC according to the dynamism of the presented face. Statistical comparisons failed to confirm an increase in IBC between interacting participants during live-online face viewing compared with static faces in Angular Gyrus, AG.

### 4.1 Online synchronous connection method

In this paper we present a general method to synchronously acquire data from physically separate laboratories. The method makes use of open-source packages which allow for convenient implementation from interested researchers. It operates using a handshake server which waits for both clients to be connected before sending a message to both clients to begin the paradigm. In this way, the server side manages the synchronisation of both clients, whilst the client-side code manages the presentation of the paradigm, triggering any equipment and storing timing information. This has the advantage of reducing the reliance on a stable internet connection because the paradigm is presented locally, and is not dependent on the other client, or the server side. It also allows each laboratory to add in their own specific requirements with respect to equipment and paradigm presentation, aiding in the ease at which it can be implemented. This current version of the method does not allow for inter-laboratory communication during the task because the client–server connection is closed after the paradigm begins, however, for future experiments examining for complex online-based interactions, the code can be extended to fulfill this requirement.

### 4.2 Face processing of live-online faces compared with static on-line faces shows increased activity in right occipital–temporal and dorsal visual stream regions

Building on the work from Zhao et al. (2023), which observed rSMG activity during the live in-person condition and not the live on-line condition, we hypothesised no difference in activity in the rSMG. Our findings support this exploratory hypothesis. In particular, activation previously observed in the supramarginal and superior temporal gyri corresponds to a lateral stream of activation thought to be of particular significance to live in-person and interactive faces (Hirsch et al., 2022; Kelley et al., 2021; Noah et al., 2020). Our findings for live on-line interactive faces differ from these prior findings. When faces are presented via a live video feed, compared with a still image, the rSMG did not display differential activity (section 3.1.1, Fig. 3 and Table 1). The literature suggests that viewing live faces compared with static (or other non-responsive representations of faces) increases activity occurring in the lateral visual stream, of which the rTPJ is a part. For example, the findings from Hirsch et al. (2022) and Noah et al. (2020) display a relative lack of activation in the rTPJ region of the brain when participants observe pre-recorded videos of faces, in contrast to faces which are live-in-person. The recent study from Zhao et al. (2023) also shows activity occurring in the rSMG for the in-person condition compared with the on-line live-face. Extending on Zhao et al. (2023), we investigated the difference in dynamic face movements between two on-line conditions, live and static, whilst maintaining physically distant participants.

Activity in the rSMG during the live-online condition relative to the in-person condition was reduced and suggests that the ability of participants to detect subtle micromovements of the face during the live-online condition may be compromised. A reduction in these socially salient signals would be expected to correspond to a reduction in neural activity. This is further reinforced by the work from Noah et al. (2020) and Hirsch et al. (2022), where activity observed during the pre-recorded face conditions is reduced relative to activity observed during live-in-person conditions in regions associated with social cognition such as the right supramarginal gyrus.

These findings are consistent with the notion that the social context of the live-online face is diminished even when contrasted against a static image, and perhaps bears a similarity to a pre-recorded face rather than a physically present, interactive, and reciprocal face. Future work is needed to compare live-online video with pre-recorded faces to further establish this.

### 4.3 Absence of evidence for coherence in live–online interactions

In order to compare with previous work relating to face processing, we computed the IBC between homologous angular gyri of interacting participants and also permuted partners. Previous work from Noah et al. (2020) and Hirsch et al. (2022) examining the IBC between during real in-person interactions and recorded video interactions display a markedly higher IBC between AG during the real person interaction compared with the recorded video. We previously hypothesised that the increased IBC between the angular gyri, AG, of interacting participants was likely due to the computational properties of that region that enabled the sharing of live facial information providing social cues. Similarly, in this study, we investigated coherence between the angular gyri of participants during viewing of live-dynamic faces when compared with viewing the static face. The analysis includes a range of frequencies consistent with the physiological haemodynamic response function expected for a 30 s task and rest period where each block is 15 s. Statistical comparisons fail to provide evidence for greater coherence in the experimental conditions than in the shuffled conditions.

## 5 Limitations

The interpretation of these results is bound by some limitations. First, the paradigm employed here was a very basic social interaction, where participants simply gazed at their partners face. Thus. interpretations about social function which relate to a typical online interaction are limited, since these interactions will normally contain some other forms of communication, such as talking to a partner, or listening to a presentation, for example. Future work could explore how the addition of other forms of social information contribute to the understanding of functional areas of the brain associated with social processes.

Further, during any condition where participants are asked to gaze at the live face of a partner with no active cognitive task may lead to differences in mindsets during the task, “mind wandering” may induce activity in other areas of the brain contributing to noise in the analysis and thus worse statistics. Here we assume that the processes are not consistent across participants and do not influence group findings. However, future work with more engaging tasks should be able to more acutely focus on the social aspects of the interaction.

With respect to the IBC, the computation of the magnitude squared coherence using wavelets has several limitations inherent in its usage (Hakim et al., 2023). In particular its inability to incorporate multimodal data sources limits the mechanistic description of the IBC, and the results pertaining to the IBC here are presented as a descriptive comparison of conditions. The general mechanisms relating to inter-brain coupling remain active areas of research and require further investigation.

Finally, our experimental setup lacked physiological monitoring equipment and short-distance channels in the optode configuration. These are of particular importance with fNIRS studies since the recorded signals are subject to systemic interference (Hakim et al., 2022; Kirilina et al., 2012; Tachtsidis & Scholkmann, 2016). We employed the Global Mean Removal method (Zhang et al., 2016) to correct for systemic interference shown to be comparable method for removing non-neuronal signal components (Noah et al., 2021). In addition to improving signal quality, recent developments in hyperscanning have shown that the inclusion of physiological monitoring can aid in the analysis of participants’ social interaction and provide an additional lens to view results through (Guglielmini et al., 2022).

## 6 Conclusions

Here we present an easy-to-use method to synchronously connect separate laboratories over the internet to conduct hyperscanning experiments using fNIRS. The method is developed using Python, a free-to-use coding language and a third computer acting as a handshake server to facilitate the synchronous presentation of stimuli, and acquisition of neural data from fNIRS systems. The method presented here can be used to examine social interactive effects as they occur on-line and IBC-at-a-distance (families separated due to work, etc.). Furthermore, it can be applied by laboratories in culturally different countries providing the opportunity to further explore on-line cross-cultural contexts. Given the increasing use of videoconferencing for teaching and working, this methodology can also be used to examine virtual learning/working environments, to improve our understanding, and to optimise how this medium of communication is employed.

We have evaluated the neural underpinnings of face processing during online face-to-face interactions. Functional activation results are presented to contrast the neural activity when participants observe a live-online face compared with a static-online face. We hypothesised that observing the live-online face will not activate regions of the brain associated with live social systems such as the right supramarginal gyrus when contrasted against observing the static faces.

Our findings are consistent with the hypothesis that regions of the brain associated with the social systems, including the rTPJ, are not more active during the observation of the live-online face than during the static-online face. We additionally evaluated the IBC of interacting participants during live-online and static-online observations of their partners faces and found no statistical evidence for increased IBC in the live-online condition. This study provides a methodological and technical framework to conduct hyperscanning studies involving online conditions and distant locations, as well as initial findings into the brain activity during online live face processing.

## Supplementary Material

Supplementary Material

## Data Availability

Data are available at https://doi.org/10.5061/dryad.2ngf1vj10 Inter laboratory synchronisation code is available at https://github.com/CogNIRS/InterLab-Connection
