# Supplementary material for: Investigating face processing in online interactions via UK–US hyperscanning using fNIRS
Source: Imaging Neurosci (Camb). 2026 Jan 23;4:IMAG.a.1101. doi: 10.1162/IMAG.a.1101 (PMC12836396; doi:10.1162/IMAG.a.1101)
Supplement: Supplementary Material [file IMAG.a.1101_supp.pdf]

Inter-Lab Synchronisation Method Connection Lag Tests

The histogram shown in Fig S1 shows the occurrence of the time differences for each connection. A time difference of 0 signifies that both client-side code is connecting at the same time, and thus the paradigms will be presented in step with each other.

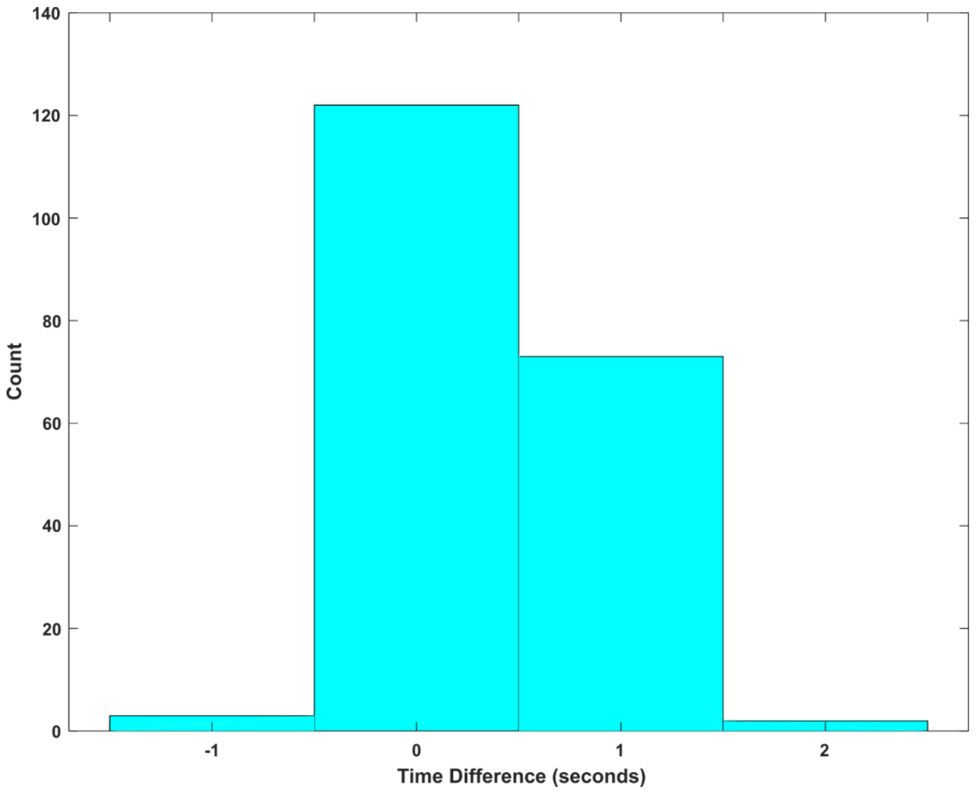

Figure S1 – Histogram showing time differences in recordings from each lab after taking into account differences in computer clock times. 200 connect-disconnect tests were run to ensure that the connections between labs was synchronous as possible. Most tests (120) were synchronous with a 0s time difference. However, 74 tests had a +1 second time lag (Yale connecting first by 1 second).

Evidently most connections occurred simultaneously, as required to ensure synchronicity between participants. However, a relatively large number of connections occurred with a 1 second lag (the Yale side connecting first). Although this is not ideal, given the temporal resolution of the haemodynamic response it is unlikely that a time difference as minimal as 1 second would have a meaningful effect on the physiological data that is recorded.

The counts presented in Fig S1 only demonstrate the differences in the connection times. These operate on the assumption that if the connection times are the same (time difference is 0) then the paradigm is presented similarly synchronously. Although this is a fair assumption because the paradigm management software is pre-loaded, and variations in compute power are taken into consideration prior to testing, it is worth testing to ensure that the time difference between paradigm presentation, and connection time is similar. Operating under the assumption that synchronous

connection time equates to synchronous paradigm presentation, the same distribution as Fig 3 would be expected. The connect-disconnect tests used to provide the histogram above was run with a comparable task to the one used in this study embedded in the client-side code. This task was composed of four pseudo-active blocks per connect-disconnect tests, resulting in 800 timing acquisitions. The same methodology as above was applied, the timings from the UCL side were subtracted from the Yale side after taking the ground truth time differences for each test start. The results are shown in Fig S2.

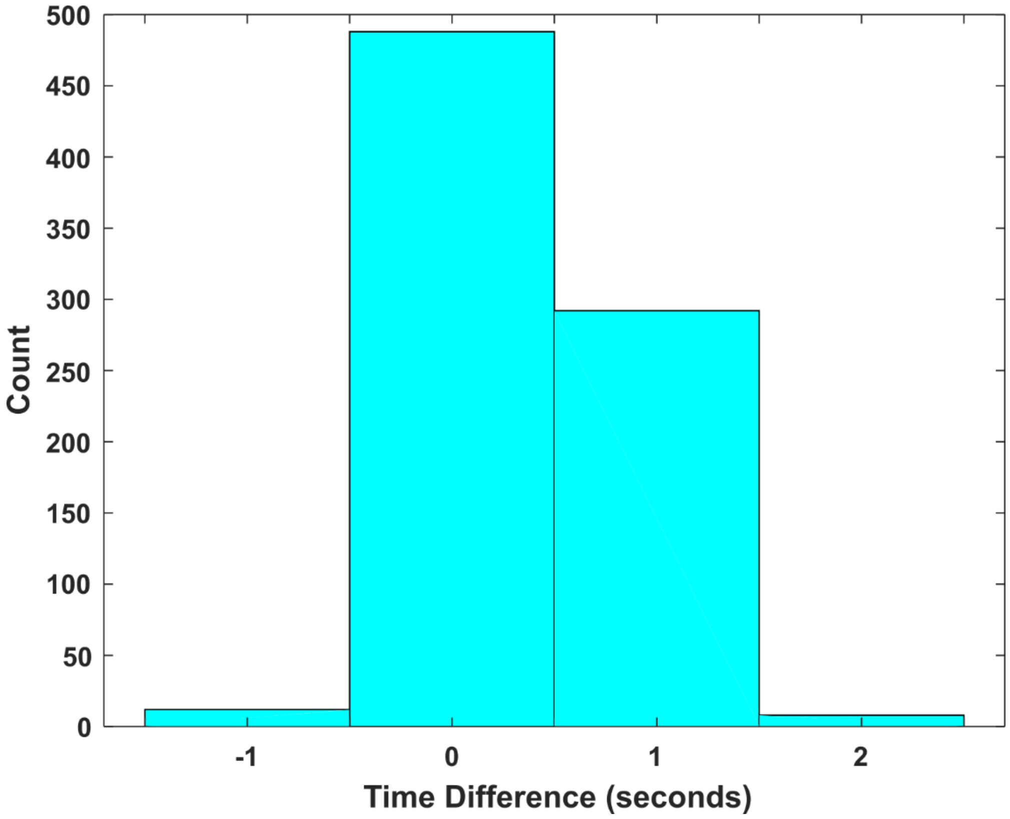

Figure S2 – Histogram showing time differences in recordings for each pseudo-task block in the connection code. As expected, the lags between connecting and disconnecting are directly related to the connect times. This supports the assumption that as long as the paradigms start at the same time, experimental stimuli will be presented simultaneously.

As expected, 4 times as many occurrences per time difference were found. Taking these two results together, the server can sufficiently manage the synchronous connection between the client computers. Furthermore, the assumption that as long as the clients connect to the server synchronously the paradigm, which is presented and managed locally, will also be synchronously presented to participants is maintained.

These simulations were conducted prior to the experiment to ensure that the occurrence of lags would be manageable. At the time of experiment, when any lags occurred and were noticeable between the labs the experiment would be stopped and restarted to maintain a zero lag stimulus presentation.

Inter-Brain Coupling analysis results

Table S1. Inter-Brain coupling Statistics between the Angular Gyri

	Real Partners		ShuffledPartners		
Period (s)	t	p	t	p	ANOVA Interaction
2.63	0.16	0.88	-0.25	0.81	F(1,76)=0.04,p=0.84
5.27	0.76	0.46	-0.98	0.34	F(1,76)=0.94,p=0.33
7.9	1.29	0.21	-0.65	0.52	F(1,76)=2.21,p=0.14
10.53	1.12	0.28	0.14	0.89	F(1,76)=1.49,p=0.23
13.16	0.8	0.44	1.42	0.17	F(1,76)=0.10,p=0.75
15.8	1.9	0.07	2.42	0.03	F(1,76)=0.85,p=0.36
18.43*	2.74	0.01*	2.11	0.05*	F(1,76)=3.04,p=0.09*
21.06	2.43	0.03	1.5	0.15	F(1,76)=3.45,p=0.07
23.69	1.88	0.08	0.77	0.45	F(1,76)=2.75,p=0.10
26.33	1.54	0.14	0.26	0.8	F(1,76)=2.07,p=0.15
28.96	1.02	0.32	-0.07	0.94	F(1,76)=1.03,p=0.31
31.59	0.23	0.82	-0.43	0.67	F(1,76)=0.11,p=0.74
34.22	-0.61	0.55	-0.67	0.51	F(1,76)=0.18,p=0.67
36.86	-1.37	0.19	-0.73	0.47	F(1,76)=1.18,p=0.28
39.49	-1.96	0.06	-0.59	0.56	F(1,76)=2.63,p=0.11

Table S1 provides the statistics for the data presented in Fig 4. Column 1 shows period (s) values representing the range of the physiological hemodynamic response function. Columns 2 and 3, Real Partners, indicate the within-in subject t statistics and levels of significance, respectively, for the comparison of the live and static face viewing conditions at increments of approximately 3 secs. Columns 4 and 5 indicate the same statistics for the control comparison where the partners are "shuffled" so that the effects of the true interaction between real partners is removed. The ANOVA Interaction columns, 6 and 7, indicate the F statistic and p value, respectively, for the interaction effect between the real and shuffled partner effects. The * at period 18.3 s and the related p-values for the related comparisons on the same line highlights the statistical comparison for the observed trend in fig 4 suggesting a possible difference between the cross-brain coherence of the rel partners during the live face and the static face conditions ($p < 0.01$). However, comparison with the shuffled control condition (based on the ANOVA interaction. $p < 0.09$) fails to support the hypothesis. We conclude, based on these statistical tests, that there is no evidence in favor of an increase in cross-brain coherence between the live face conditions and the statics face condition for these on-line interactions.
